# Poly[[tetraaqua­bis(μ_3_-1*H*-benzimidazole-5,6-dicarboxyl­ato)dicobalt(II)] trihydrate]

**DOI:** 10.1107/S1600536809049083

**Published:** 2009-11-21

**Authors:** Jun-Dan Fu, Zhi-Wei Tang, Ming-Yue Yuan, Yi-Hang Wen

**Affiliations:** a Zhejiang Key Laboratory for Reactive Chemistry on Solid Surfaces, Institute of Physical Chemistry, Zhejiang Normal University, Jinhua, Zhejiang 321004, People’s Republic of China

## Abstract

The title complex, {[Co_2_(C_9_H_4_N_2_O_4_)_2_(H_2_O)_4_]·3H_2_O}_*n*_, was synthesized hydro­thermally. The unique Co^II^ ion is coordin­ated in a distorted octa­hedral coordination environment by two water mol­ecules and three symmetry-related 1*H*-benzimid­azole-5,6-dicarboxyl­ate (Hbidc) ligands. The Hbidc ligands coordinate *via* a bis-chelating and mono-chelating carboxyl­ate group and by an imidazole group N atom, bridging the Co^II^ ions and forming an extended two-dimensional structure in the *ab* plane. In the crystal structure, inter­molecular N—H⋯O and O—H⋯O hydrogen bonds connect complex and solvent water mol­ecules, forming a three-dimensional supermolecular network. One of the solvent water mol­ecules lies on a twofold rotation axis.

## Related literature

For background information on carboxyl­ate ligands in coordination chemistry, see: Laduca (2009 ▶); Grodzicki *et al.* (2005 ▶). For the isostructural Ni(II) complex, see: Yao *et al.* (2008 ▶). For related structures, see: Wei *et al.* (2008 ▶); Xu & Yu (2009 ▶).
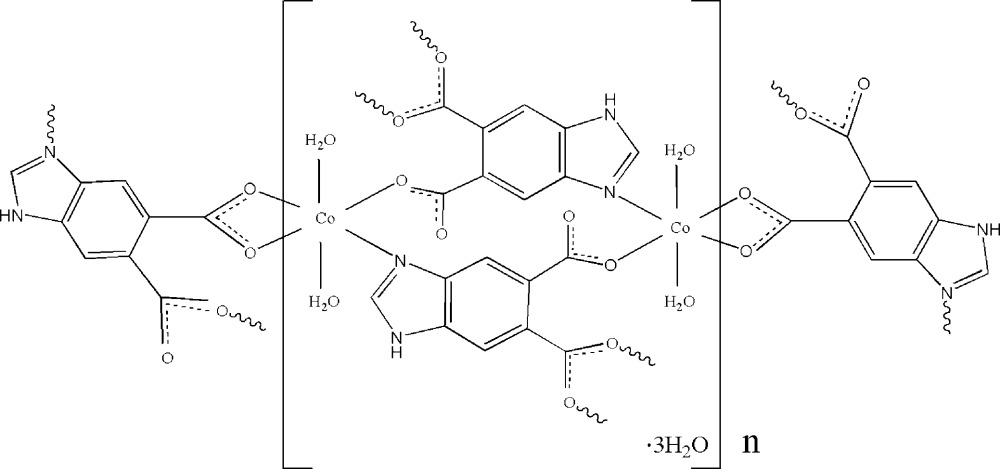


## Experimental

### 

#### Crystal data

[Co_2_(C_9_H_4_N_2_O_4_)_2_(H_2_O)_4_]·3H_2_O
*M* *_r_* = 652.26
Monoclinic, 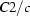

*a* = 22.4085 (18) Å
*b* = 9.1564 (7) Å
*c* = 13.0907 (10) Å
β = 121.006 (4)°
*V* = 2302.2 (3) Å^3^
*Z* = 4
Mo *K*α radiation
μ = 1.53 mm^−1^
*T* = 296 K
0.43 × 0.25 × 0.07 mm

#### Data collection

Bruker APEXII diffractometer
Absorption correction: multi-scan (*SADABS*; Sheldrick, 1996 ▶) *T*_min_ = 0.63, *T*_max_ = 0.90
9315 measured reflections
2656 independent reflections
2402 reflections with *I* > 2σ(*I*)
*R*_int_ = 0.018

#### Refinement

*R*[*F* ^2^ > 2σ(*F* ^2^)] = 0.029
*wR*(*F* ^2^) = 0.087
*S* = 1.03
2656 reflections
174 parameters
6 restraints
H-atom parameters constrained
Δρ_max_ = 0.81 e Å^−3^
Δρ_min_ = −0.63 e Å^−3^

### 

Data collection: *APEX2* (Bruker, 2006 ▶); cell refinement: *SAINT* (Bruker, 2006 ▶); data reduction: *SAINT*; program(s) used to solve structure: *SHELXS97* (Sheldrick, 2008 ▶); program(s) used to refine structure: *SHELXL97* (Sheldrick, 2008 ▶); molecular graphics: *SHELXTL* (Sheldrick, 2008 ▶); software used to prepare material for publication: *SHELXTL*.

## Supplementary Material

Crystal structure: contains datablocks I, global. DOI: 10.1107/S1600536809049083/lh2948sup1.cif

Structure factors: contains datablocks I. DOI: 10.1107/S1600536809049083/lh2948Isup2.hkl

Additional supplementary materials:  crystallographic information; 3D view; checkCIF report

## Figures and Tables

**Table 1:** Hydrogen-bond geometry (Å, °)

*D*—H⋯*A*	*D*—H	H⋯*A*	*D*⋯*A*	*D*—H⋯*A*
O3*W*—H3*WA*⋯O1^i^	0.84	1.97	2.798 (2)	170
N1—H1*A*⋯O1*W* ^i^	0.86	2.45	3.130 (2)	136
O2*W*—H2*WA*⋯O4^i^	0.84	1.99	2.786	157
O3*W*—H3*WB*⋯O3^ii^	0.84	1.85	2.641 (2)	158
O4*W*—H4*WB*⋯O3^iii^	0.84	2.60	3.095 (2)	119
O4*W*—H4*WA*⋯O2^iii^	0.84	1.86	2.679 (2)	165
O4*W*—H4*WB*⋯O2*W* ^iv^	0.84	2.02	2.769	148
N1—H1*A*⋯O2*W*	0.86	2.31	2.899	125
O1*W*—H1*W*⋯O3^v^	0.84	1.98	2.8218 (18)	180
O2*W*—H2*WB*⋯O3*W* ^vi^	0.84	2.16	2.949	157

Symmetry codes: (i) 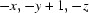; (ii) 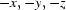; (iii) 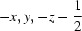; (iv) 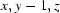; (v) 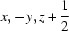; (vi) 
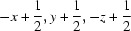
.

**Fig. 1.**
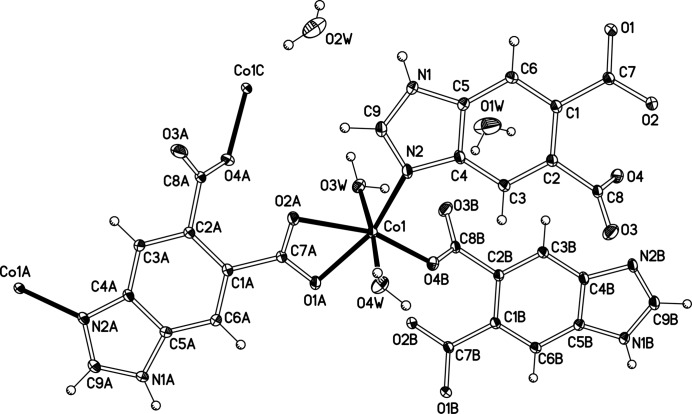
Part of the 2-D structure of (I), showing the atom-numbering scheme. Displacement ellipsoids are drawn at the 30% probability level. [Symmetry codes: (A)-x, -y, -z; (B)x + 1/2, -y + 1/2, z + 1/2; (c)x - 1/2,-y + 1/2, z - 1/2.

**Fig. 2.**
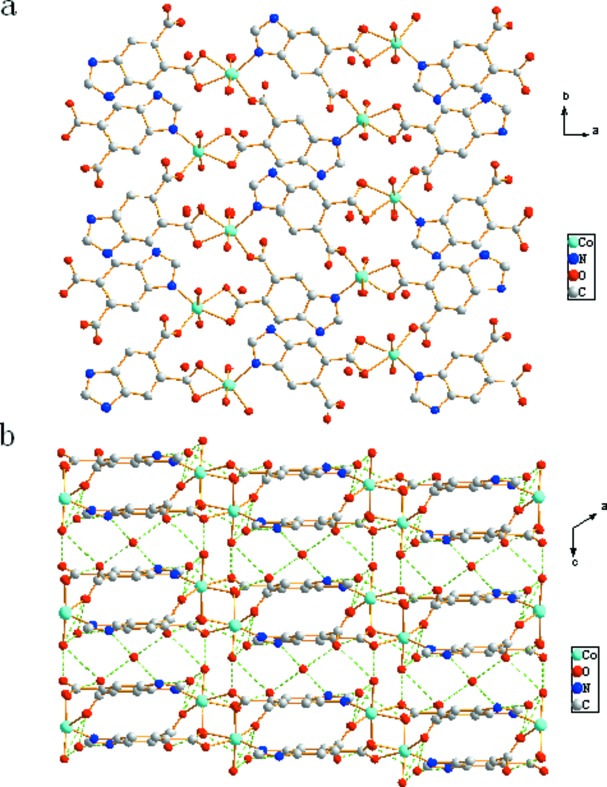
(a) Part of the 2-D structure of (I) viewed along the crystallographic c-axis. (b) Part of the crystal structure showing the donor···acceptor atom distances of hydrogen bonds as dashed lines.

## supplementary crystallographic information

### Comment

It is well known that carboxylate ligands play an important role in coordination chemistry (Grodzicki *et al. *, 2005; Laduca, 2009). In recent years, the interaction of Hbidc with several metal ions has been studied, due to its unique ability to form stable chelates in diverse coordination modes such as bidentate, meridian and bridging (Wei *et al.*, 2008; Yao *et al.*, 2008). Herein we report the synthesis and crystal structure of the title two-dimensional complex of Hbidc (I).

Part of the 2-D structure of (I) is shown in Fig.1. The unique Co^II^ ion is six-coordinated by one N atom and three O atoms from from three symmetry related Hbidc ligands and two oxygen atoms from two water molecules. Each Hbidc ligand coordinates via a chelating carboxylate group and a single oxygen atom of another carboxylate group bridging two Co^II^ ions to form a one-dimensional chain along the *b*-axis with a Co···Co separation of 5.4374 (5) Å. In addition, Hbidc ligands coordinate through a N atom to connect the adjacent chains forming a two-dimensional network with chains separated by ca. 7.06 Å (see Fig. 2a). In the crystal structure, intermolecular N-H···O and O-H···O hydrogen bonds connect complex and solvent water molecules to form a three-dimensional supermolecular network (see Table 1 and Fig. 2b).

### Experimental

A mixture of CoSO_4_.7H_2_O(0.141 g, 0.5 mmol), benzimidazole-5,6-dicarboxylic acid(0.103 g, 0.5 mmol), H_2_O(16 ml)and 4-sulfophthalic(1 ml)(solution pH = 5) was sealed in a 25 ml Teflon-lined stainless steel reactor and heated at 393 k for 3 d. On completion of the reaction, the reactor was cooled slowly to room temperature and the mixture was filtered, giving red single crystals suitable for X-ray analysis in 30% yield.

### Refinement

H-atoms were positioned geometrically and included in the refinement using a riding-model approximation [C–H = 0.93, O–H = 0.84 and N-H = 0.86Å] with *U*_iso_(H) = 1.2*U*_eq_(C,N) or 1.5*U*_eq_(O).

### Crystal data

**Table:**

[Co_2_(C_9_H_4_N_2_O_4_)_2_(H_2_O)_4_]·3H_2_O	*F*(000) = 1328
*M**_r_* = 652.26	*D*_x_ = 1.882 Mg m^−^^3^
Monoclinic, *C*2/*c*	Mo *K*α radiation, λ = 0.71073 Å
Hall symbol: -C 2yc	Cell parameters from 5695 reflections
*a* = 22.4085 (18) Å	θ = 2.1–27.6°
*b* = 9.1564 (7) Å	µ = 1.53 mm^−^^1^
*c* = 13.0907 (10) Å	*T* = 296 K
β = 121.006 (4)°	Block, red
*V* = 2302.2 (3) Å^3^	0.43 × 0.25 × 0.07 mm
*Z* = 4	

### Data collection

**Table:**

Bruker APEXII diffractometer	2656 independent reflections
Radiation source: fine-focus sealed tube	2402 reflections with *I* > 2σ(*I*)
graphite	*R*_int_ = 0.018
ω scans	θ_max_ = 27.6°, θ_min_ = 2.1°
Absorption correction: multi-scan (*SADABS*; Sheldrick, 1996)	*h* = −29→27
*T*_min_ = 0.63, *T*_max_ = 0.90	*k* = −11→11
9315 measured reflections	*l* = −16→17

### Refinement

**Table:**

Refinement on *F*^2^	Primary atom site location: structure-invariant direct methods
Least-squares matrix: full	Secondary atom site location: difference Fourier map
*R*[*F*^2^ > 2σ(*F*^2^)] = 0.029	Hydrogen site location: inferred from neighbouring sites
*wR*(*F*^2^) = 0.087	H-atom parameters constrained
*S* = 1.03	*w* = 1/[σ^2^(*F*_o_^2^) + (0.0533*P*)^2^ + 3.4124*P*] where *P* = (*F*_o_^2^ + 2*F*_c_^2^)/3
2656 reflections	(Δ/σ)_max_ = 0.001
174 parameters	Δρ_max_ = 0.81 e Å^−^^3^
6 restraints	Δρ_min_ = −0.63 e Å^−^^3^

### Special details

**Table:**

Geometry. All e.s.d.'s (except the e.s.d. in the dihedral angle between two l.s. planes) are estimated using the full covariance matrix. The cell e.s.d.'s are taken into account individually in the estimation of e.s.d.'s in distances, angles and torsion angles; correlations between e.s.d.'s in cell parameters are only used when they are defined by crystal symmetry. An approximate (isotropic) treatment of cell e.s.d.'s is used for estimating e.s.d.'s involving l.s. planes.
Refinement. Refinement of *F*^2^ against ALL reflections. The weighted *R*-factor *wR* and goodness of fit *S* are based on *F*^2^, conventional *R*-factors *R* are based on *F*, with *F* set to zero for negative *F*^2^. The threshold expression of *F*^2^ > σ(*F*^2^) is used only for calculating *R*-factors(gt) *etc*. and is not relevant to the choice of reflections for refinement. *R*-factors based on *F*^2^ are statistically about twice as large as those based on *F*, and *R*- factors based on ALL data will be even larger.

### Fractional atomic coordinates and isotropic or equivalent isotropic displacement parameters (Å^2^)

**Table:**

	*x*	*y*	*z*	*U*_iso_*/*U*_eq_	
Co1	0.199951 (12)	0.14498 (3)	0.12124 (2)	0.01825 (10)	
O1	−0.19686 (7)	0.44398 (15)	−0.25564 (13)	0.0239 (3)	
O2	−0.20916 (7)	0.22564 (15)	−0.33254 (12)	0.0216 (3)	
O3	−0.10064 (8)	−0.07292 (17)	−0.23544 (16)	0.0335 (4)	
O3W	0.20211 (8)	0.25089 (16)	0.26967 (13)	0.0277 (3)	
H3WA	0.1974	0.3419	0.2690	0.042*	
H3WB	0.1706	0.2071	0.2742	0.042*	
O4	−0.14121 (7)	0.02445 (15)	−0.12688 (12)	0.0224 (3)	
O4W	0.20838 (8)	0.03724 (17)	−0.01154 (13)	0.0302 (3)	
H4WA	0.2019	0.0885	−0.0696	0.045*	
H4WB	0.1863	−0.0415	−0.0365	0.045*	
N1	0.07455 (8)	0.50513 (18)	−0.06630 (15)	0.0222 (3)	
H1A	0.0716	0.5976	−0.0794	0.027*	
N2	0.12237 (8)	0.28672 (18)	0.00087 (15)	0.0225 (3)	
C1	−0.09320 (9)	0.3057 (2)	−0.19386 (16)	0.0181 (4)	
C2	−0.06298 (9)	0.1649 (2)	−0.15733 (16)	0.0175 (4)	
C3	0.00868 (10)	0.1468 (2)	−0.09321 (18)	0.0207 (4)	
H3A	0.0285	0.0544	−0.0700	0.025*	
C4	0.05016 (9)	0.2710 (2)	−0.06466 (17)	0.0195 (4)	
C5	0.01982 (10)	0.4091 (2)	−0.10514 (17)	0.0189 (4)	
C6	−0.05185 (9)	0.4293 (2)	−0.16866 (17)	0.0195 (4)	
H6A	−0.0714	0.5216	−0.1933	0.023*	
C7	−0.17032 (9)	0.3262 (2)	−0.26428 (16)	0.0177 (3)	
C8	−0.10569 (9)	0.0287 (2)	−0.17727 (16)	0.0194 (4)	
C9	0.13324 (10)	0.4278 (2)	−0.00439 (18)	0.0240 (4)	
H9A	0.1774	0.4691	0.0315	0.029*	
O1W	0.0000	0.24202 (17)	0.2500	0.1066 (18)	
H1W	−0.0300	0.1917	0.2543	0.160*	
O2W	0.1643	0.75912 (17)	0.0016	0.0530 (5)	
H2WA	0.1492	0.8072	0.0382	0.080*	
H2WB	0.2053	0.7403	0.0558	0.080*	

### Atomic displacement parameters (Å^2^)

**Table:**

	*U*^11^	*U*^22^	*U*^33^	*U*^12^	*U*^13^	*U*^23^
Co1	0.01193 (15)	0.01637 (16)	0.02247 (16)	−0.00183 (8)	0.00601 (11)	0.00077 (9)
O1	0.0152 (6)	0.0189 (7)	0.0326 (7)	0.0021 (5)	0.0086 (6)	−0.0031 (6)
O2	0.0145 (6)	0.0201 (7)	0.0250 (7)	−0.0001 (5)	0.0064 (5)	−0.0035 (5)
O3	0.0321 (8)	0.0251 (8)	0.0530 (10)	−0.0099 (6)	0.0289 (8)	−0.0140 (7)
O3W	0.0269 (8)	0.0208 (7)	0.0381 (8)	−0.0053 (6)	0.0186 (7)	−0.0047 (6)
O4	0.0197 (6)	0.0207 (7)	0.0290 (7)	−0.0043 (5)	0.0139 (6)	−0.0005 (5)
O4W	0.0398 (9)	0.0241 (7)	0.0287 (7)	−0.0068 (6)	0.0191 (7)	−0.0012 (6)
N1	0.0166 (8)	0.0159 (8)	0.0313 (9)	−0.0036 (6)	0.0104 (7)	−0.0003 (6)
N2	0.0118 (7)	0.0221 (8)	0.0271 (8)	−0.0023 (6)	0.0054 (6)	0.0023 (7)
C1	0.0134 (8)	0.0187 (9)	0.0201 (8)	0.0002 (7)	0.0072 (7)	0.0008 (7)
C2	0.0138 (8)	0.0164 (9)	0.0202 (8)	−0.0008 (6)	0.0072 (7)	0.0011 (7)
C3	0.0148 (9)	0.0162 (9)	0.0263 (9)	0.0010 (6)	0.0072 (7)	0.0029 (7)
C4	0.0123 (8)	0.0212 (9)	0.0219 (9)	−0.0009 (7)	0.0067 (7)	0.0021 (7)
C5	0.0171 (9)	0.0161 (9)	0.0225 (9)	−0.0024 (7)	0.0095 (7)	0.0000 (7)
C6	0.0163 (8)	0.0159 (9)	0.0244 (9)	0.0015 (7)	0.0091 (7)	0.0023 (7)
C7	0.0135 (8)	0.0176 (8)	0.0208 (9)	0.0006 (7)	0.0079 (7)	0.0026 (7)
C8	0.0119 (8)	0.0173 (9)	0.0237 (9)	−0.0013 (7)	0.0054 (7)	0.0017 (7)
C9	0.0150 (9)	0.0241 (10)	0.0289 (10)	−0.0037 (7)	0.0084 (8)	−0.0001 (8)
O1W	0.103 (3)	0.0399 (18)	0.247 (6)	0.000	0.140 (4)	0.000
O2W	0.0709 (14)	0.0310 (10)	0.0514 (11)	−0.0099 (9)	0.0275 (10)	−0.0037 (8)

### Geometric parameters (Å, °)

**Table:**

Co1—O4^i^	2.0603 (14)	N1—C5	1.376 (2)
Co1—N2	2.0898 (16)	N1—H1A	0.8598
Co1—O4W	2.0901 (15)	N2—C9	1.322 (3)
Co1—O3W	2.1497 (15)	N2—C4	1.395 (2)
Co1—O2^ii^	2.1560 (13)	C1—C6	1.390 (3)
Co1—O1^ii^	2.1837 (14)	C1—C2	1.420 (3)
Co1—C7^ii^	2.5057 (18)	C1—C7	1.493 (2)
O1—C7	1.265 (2)	C2—C3	1.386 (3)
O1—Co1^iii^	2.1837 (14)	C2—C8	1.511 (2)
O2—C7	1.266 (2)	C3—C4	1.393 (3)
O2—Co1^iii^	2.1560 (13)	C3—H3A	0.9300
O3—C8	1.244 (2)	C4—C5	1.404 (3)
O3W—H3WA	0.8399	C5—C6	1.389 (3)
O3W—H3WB	0.8399	C6—H6A	0.9300
O4—C8	1.269 (2)	C7—Co1^iii^	2.5057 (18)
O4—Co1^i^	2.0603 (14)	C9—H9A	0.9300
O4W—H4WA	0.8401	O1W—H1W	0.8401
O4W—H4WB	0.8393	O2W—H2WA	0.8400
N1—C9	1.338 (3)	O2W—H2WB	0.8401
			
O4^i^—Co1—N2	101.30 (6)	C9—N2—Co1	122.80 (13)
O4^i^—Co1—O4W	90.43 (6)	C4—N2—Co1	130.58 (13)
N2—Co1—O4W	93.58 (7)	C6—C1—C2	121.02 (16)
O4^i^—Co1—O3W	91.41 (6)	C6—C1—C7	117.55 (16)
N2—Co1—O3W	91.38 (6)	C2—C1—C7	121.40 (16)
O4W—Co1—O3W	174.27 (6)	C3—C2—C1	121.02 (16)
O4^i^—Co1—O2^ii^	158.80 (5)	C3—C2—C8	116.03 (16)
N2—Co1—O2^ii^	99.73 (6)	C1—C2—C8	122.80 (16)
O4W—Co1—O2^ii^	90.90 (6)	C2—C3—C4	118.00 (17)
O3W—Co1—O2^ii^	85.43 (5)	C2—C3—H3A	121.0
O4^i^—Co1—O1^ii^	98.39 (5)	C4—C3—H3A	121.0
N2—Co1—O1^ii^	160.30 (6)	C3—C4—N2	130.70 (18)
O4W—Co1—O1^ii^	85.57 (6)	C3—C4—C5	120.52 (17)
O3W—Co1—O1^ii^	88.79 (6)	N2—C4—C5	108.77 (16)
O2^ii^—Co1—O1^ii^	60.64 (5)	N1—C5—C6	132.21 (18)
O4^i^—Co1—C7^ii^	128.59 (6)	N1—C5—C4	105.67 (16)
N2—Co1—C7^ii^	130.06 (6)	C6—C5—C4	122.11 (17)
O4W—Co1—C7^ii^	88.46 (6)	C5—C6—C1	117.23 (17)
O3W—Co1—C7^ii^	86.15 (6)	C5—C6—H6A	121.4
O2^ii^—Co1—C7^ii^	30.33 (6)	C1—C6—H6A	121.4
O1^ii^—Co1—C7^ii^	30.31 (6)	O1—C7—O2	119.96 (16)
C7—O1—Co1^iii^	89.07 (11)	O1—C7—C1	119.97 (16)
C7—O2—Co1^iii^	90.30 (11)	O2—C7—C1	120.07 (16)
Co1—O3W—H3WA	119.7	O1—C7—Co1^iii^	60.62 (9)
Co1—O3W—H3WB	102.7	O2—C7—Co1^iii^	59.36 (9)
H3WA—O3W—H3WB	111.6	C1—C7—Co1^iii^	178.35 (14)
C8—O4—Co1^i^	128.68 (13)	O3—C8—O4	125.06 (18)
Co1—O4W—H4WA	116.2	O3—C8—C2	118.29 (17)
Co1—O4W—H4WB	116.2	O4—C8—C2	116.53 (17)
H4WA—O4W—H4WB	109.6	N2—C9—N1	113.50 (17)
C9—N1—C5	107.24 (16)	N2—C9—H9A	123.3
C9—N1—H1A	126.4	N1—C9—H9A	123.3
C5—N1—H1A	126.4	H2WA—O2W—H2WB	102.3
C9—N2—C4	104.80 (16)		
			
O4^i^—Co1—N2—C9	158.93 (16)	C3—C4—C5—N1	−177.40 (18)
O4W—Co1—N2—C9	−109.92 (17)	N2—C4—C5—N1	1.9 (2)
O3W—Co1—N2—C9	67.21 (17)	C3—C4—C5—C6	3.7 (3)
O2^ii^—Co1—N2—C9	−18.39 (18)	N2—C4—C5—C6	−177.00 (18)
O1^ii^—Co1—N2—C9	−23.1 (3)	N1—C5—C6—C1	179.8 (2)
C7^ii^—Co1—N2—C9	−18.9 (2)	C4—C5—C6—C1	−1.6 (3)
O4^i^—Co1—N2—C4	−3.25 (19)	C2—C1—C6—C5	−0.9 (3)
O4W—Co1—N2—C4	87.90 (18)	C7—C1—C6—C5	−178.77 (17)
O3W—Co1—N2—C4	−94.97 (18)	Co1^iii^—O1—C7—O2	1.72 (18)
O2^ii^—Co1—N2—C4	179.43 (17)	Co1^iii^—O1—C7—C1	−178.23 (15)
O1^ii^—Co1—N2—C4	174.76 (16)	Co1^iii^—O2—C7—O1	−1.74 (18)
C7^ii^—Co1—N2—C4	178.90 (16)	Co1^iii^—O2—C7—C1	178.21 (15)
C6—C1—C2—C3	1.4 (3)	C6—C1—C7—O1	−30.8 (3)
C7—C1—C2—C3	179.21 (18)	C2—C1—C7—O1	151.34 (18)
C6—C1—C2—C8	176.81 (17)	C6—C1—C7—O2	149.27 (18)
C7—C1—C2—C8	−5.4 (3)	C2—C1—C7—O2	−28.6 (3)
C1—C2—C3—C4	0.6 (3)	Co1^i^—O4—C8—O3	0.8 (3)
C8—C2—C3—C4	−175.08 (17)	Co1^i^—O4—C8—C2	−175.08 (12)
C2—C3—C4—N2	177.8 (2)	C3—C2—C8—O3	−62.2 (2)
C2—C3—C4—C5	−3.1 (3)	C1—C2—C8—O3	122.2 (2)
C9—N2—C4—C3	177.6 (2)	C3—C2—C8—O4	114.0 (2)
Co1—N2—C4—C3	−17.9 (3)	C1—C2—C8—O4	−61.7 (2)
C9—N2—C4—C5	−1.6 (2)	C4—N2—C9—N1	0.7 (2)
Co1—N2—C4—C5	162.99 (14)	Co1—N2—C9—N1	−165.38 (14)
C9—N1—C5—C6	177.3 (2)	C5—N1—C9—N2	0.4 (2)
C9—N1—C5—C4	−1.4 (2)		

Symmetry codes: (i) −*x*, −*y*, −*z*; (ii) *x*+1/2, −*y*+1/2, *z*+1/2; (iii) *x*−1/2, −*y*+1/2, *z*−1/2.

### Hydrogen-bond geometry (Å, °)

**Table:**

*D*—H···*A*	*D*—H	H···*A*	*D*···*A*	*D*—H···*A*
O3W—H3WA···O1^iv^	0.84	1.97	2.798 (2)	170
N1—H1A···O1W^iv^	0.86	2.45	3.130 (2)	136
O2W—H2WA···O4^iv^	0.84	1.99	2.78546	157
O3W—H3WB···O3^i^	0.84	1.85	2.641 (2)	158
O4W—H4WB···O3^v^	0.84	2.60	3.095 (2)	119
O4W—H4WA···O2^v^	0.84	1.86	2.679 (2)	165
O4W—H4WB···O2W^vi^	0.84	2.02	2.76864	148
N1—H1A···O2W	0.86	2.31	2.89857	125
O1W—H1W···O3^vii^	0.84	1.98	2.8218 (18)	180
O2W—H2WB···O3W^viii^	0.84	2.16	2.94922	157

Symmetry codes: (iv) −*x*, −*y*+1, −*z*; (i) −*x*, −*y*, −*z*; (v) −*x*, *y*, −*z*−1/2; (vi) *x*, *y*−1, *z*; (vii) *x*, −*y*, *z*+1/2; (viii) −*x*+1/2, *y*+1/2, −*z*+1/2.
